# Assessment of E/A ratio helps emergency clinicians in the management of patients with acute dyspnea

**DOI:** 10.1007/s11739-023-03279-8

**Published:** 2023-04-27

**Authors:** Maria Immacolata Arnone, Alfonso Sforza, Maria Viviana Carlino, Mario Guarino, Riccardo Candido, Dario Bertolone, Ilaria Fucile, Nicola De Luca, Costantino Mancusi

**Affiliations:** 1 Emergency Department, CTO Hospital, Naples, Italy; 2 Cardiology Department and Cardiology Intensive Care Unit, Santa Maria Delle Grazie, Pozzuoli, Italy; 3 Cardiology Department and Cardiology Intensive Care Unit, San Giuliano Hospital, Giugliano, Italy; 4 Emergency Medicine School & Department of Advanced Biomedical Science, Federico II University Hospital, Via Sergio Pansini 5, 80131 Naples, Italy

**Keywords:** Lung ultrasound, Focused cardiac ultrasonography, Multi-organ ultrasound, Diastolic function, Emergency department

## Abstract

Acute dyspnea (AD) is one of the main reasons for admission to the Emergency Department (ED). In the last years integrated ultrasound examination (IUE) of lung, heart and inferior vena cava (IVC) has become an extension of clinical examination for a fast differential diagnosis. The aim of present study is to assess the feasibility and diagnostic accuracy of E/A ratio for diagnosing acute heart failure (aHF) in patients with acute dyspnea. We included 92 patients presenting to the ED of CTO Hospital in Naples (Italy) for AD. All patients underwent IUE of lung-heart-IVC with a portable ultrasound device. Left ventricle diastolic function was assessed using pulse wave doppler at the tips of the mitral valve and E wave velocity and E/A ratio were recorded. The FINAL diagnosis was determined by two expert reviewers: acute HF or non-acute HF (non-aHF). We used 2 × 2 contingency tables to analyze sensitivity, specificity, positive predictive and negative predictive value of ultrasound parameters for the diagnosis of AD, comparing with the FINAL diagnosis. Lung ultrasound (LUS) showed high sensitivity, good specificity and accuracy in identification of patients with aHF. However, the highest accuracy was obtained by diastolic function parameters. The E/A ratio showed the highest diagnostic performance with an AUC for aHF of 0.93. In patients presenting with AD, E/A ratio is easy to obtain in a fast ultrasound protocol and showed an excellent accuracy for diagnosis of aHF.

## Introduction

Initial assessment of patients with acute dyspnea is commonly based on medical history, physical examination, chest X-ray, 12-lead electrocardiogram, arterial blood gas analysis and routine blood tests [1].

In the last years, integrated ultrasound examination (IUE) of lung, heart and inferior vena cava become an extension of clinical examination for a fast assessment of acute dyspnea in emergency setting [2–4].

Lung ultrasound (LUS) is widely demonstrated to be superior for the identification and evaluation of extravascular lung water when compared to chest X-ray [5–7]. The implementation of LUS with clinical assessment improves the diagnostic accuracy for acute Heart Failure (aHF) in the context of emergency department [8–10]. Focused cardiac ultrasonography (FoCUS) is a non-invasive and reproducible examination that has a great diagnostic and prognostic value in patients with aHF [11, 12].

We have recently demonstrated that the combination of positive LUS with FoCUS, including dilated left atrium and reduced ejection fraction, substantially extends the spectrum of recognizable aHF [13].

Left ventricle (LV) diastolic dysfunction represents the main pathophysiological abnormalities in HF as it plays an important role in determining increased LV filling pressures with consequent development of venous congestion and pulmonary edema. Its assessment in the context of emergency department is feasible and may help to improve rapid diagnostic assessment of patients with acute dyspnea [14].

The aim of the present study is to assess feasibility and diagnostic accuracy of simple parameter of diastolic function assessment (E wave velocity and E/A ratio) included in a fast protocol of IUE for diagnosing aHF in patients with undifferentiated dyspnea in emergency department.

## Materials and methods

From January 2019 to June 2019, we enrolled 113 patients presenting to the emergency department of CTO Hospital in Naples (Italy) for acute dyspnea or sudden worsening of chronic dyspnea within the previous 48 h. Patients with dyspnea of traumatic origin were excluded. 21 patients with atrial fibrillation (AF) were excluded from the study; thus, the final study population included 92 patients. Patients were enrolled consecutively during the shift of the involved physicians in the study. All patients underwent clinical examination, blood gas analysis, chest X-ray, ECG, and routine blood tests (including BNP). BNP was considered negative if < 100 pg/mL according to current guidelines on HF [15]. Chest X-ray was considered positive when detected interstitial syndrome and/or the presence of pleural effusion. All patients underwent IUE with a portable ultrasound device using convex probe for LUS and cardiac probe for heart and inferior vena cava evaluation. An emergency physician with good experience of LUS and transthoracic echocardiography, who was not taking care of the patient, performed IUE with the patient in semi-sitting or supine position. The emergency physician performing ultrasound examination have received a dedicated training which included: 3 months of echocardiography training in outpatients clinic with 150 echocardiographic and LUS examinations with a wide variety of pathologic conditions and 50 examination performed in emergency department under close supervision. For LUS was followed a simplified protocol that provides two scans at each side, anteriorly on the II intercostal space, mid-clavicular line, and lateral on the V intercostal space, mid-axillary line, to sample upper and lower lungs using a convex probe (3.5–5 MHz, abdominal pre-set) [5, 16]. The presence or the absence of interstitial syndrome (IS, defined as the presence of at least 3 B lines for lung field) and the presence or the absence of pleural effusion (defined as hypo-anechoic space between the parietal and visceral pleura) was evaluated. Lung ultrasound was defined positive for bilateral IS and/or effusion if IS and/or effusion was present at least in the lateral scan (V intercostal space, mid-axillary line) bilaterally [17–19]. FoCUS examination included two projections (parasternal long-axis view and apical view). Ejection fraction (EF) was estimated visually and categorized as preserved/mildly reduced if > 40% or severely reduced if ≤ 40% (HFrEF), based on cut-off point provided by the ESC guidelines [20]. Left atrium was considered dilated if anteroposterior diameter (in the parasternal long-axis view) was visually estimated to be > 4 cm in both genders [13]. The IVC was evaluated in subcostal view for the presence of dilatation (visually estimated to be > 2 cm) and hypo-reactivity with breathing (variation of size < 50% about) [21]. LV diastolic function was evaluated using pulse wave doppler at the tips of the opened mitral valve in 4-chamber apical view and E wave velocity and E/A ratio were recorded. Three consecutive cycles at end expiration were recorded, and the average E/A ratio was registered. [20, 22, 23].

The ultrasound examination was done within 30 min from the arrival of the patients in ED. Two emergency physicians performed all the ultrasound examinations. The final diagnosis, considered as the gold standard, was issued by two independent observers who had access to the entire medical chart (from Emergency Department admission to hospital discharge) of each patient (medical history, clinical examination, blood gas analysis, CXR, ECG, and routine blood tests (including BNP). Based on this revision, patients were classified into two groups: aHF or non-cardiac dyspnea (non-aHF). Acute HF was defined according to current guidelines [20]. In patients with coexistence of heart failure and another cause of dyspnea, the main diagnosis was aHF [24, 25]. Informed consent was obtained from each patient included in the study and follows the principles of the Declaration of Helsinki. The study was approved by the ethics committee of the University of Naples Federico II. Data were analyzed using SPSS version 24.0 (SPSS, Chicago, IL, USA). Continuous data are expressed as mean ± 1 standard deviation and categorical variables as percentages. Quantitative variables were compared using Student’s *t*-test, while chi-square distribution was used to compare categorical variables. A *p*-value < 0.05 was considered statistically significant. The population was divided into two groups: patients with aHF and patients without acute HF (non-aHF). The performance of each different diagnostic tests for the diagnosis of aHF (chest X-ray, BNP, bilateral IS and/or effusion, dilated left atrium, and EF ≤ 40%, E/A ratio and E wave) was analyzed, and comparisons were made among them, using sensitivity, specificity, positive predictive value, negative predictive value. Confidence intervals (CI) at 95% were calculated for sensitivity, specificity, positive predictive value, and negative predictive value. Areas under the curve (AUC) and receiver operating characteristic (ROC) curve were used to compare the performance of each different ultrasound diagnostic tests in relation to the final diagnosis [26]*.* Yuoden index was used to calculate the optimal threshold value (cut-off point) for E/A ratio to identify patients with acute HF. A combined clinical score was built to assess the diagnostic accuracy of E/A ratio on top of common clinical characteristics of the patients including anamnestic history, chest X ray, BNP value and dilated LA. We estimated probability of having aHF as final diagnosis using a multivariable logistic regression model including anamnestic history, chest X ray, BNP value and dilated LA. Then, in a second model, E/A ratio was also added. For each model, individual risk estimates were based on the sum of weighted scores for each variable and compared using receiving operating characteristic curves, and the areas under the curve (AUC) were calculated. Detection of a significant difference between two AUC indicates significant difference in the overall ability of the prediction with the largest area indicating the best predictive model. The method of Delong et al [26] for the calculation of the Standard Error of the AUC was used to compare diagnostic accuracy of different modalities. For comparison of sensitivity and specificity McNemar test was used with STATA version 14.

## Results

Our study population included 92 patients 25 patients (27%) had aHF, and 67 patients (73%) had non-cardiac dyspnea (non-AHF). Number of patients with HFrEF was 13, first episode of HF was diagnosed in 13 patients. Table 1 displays the final diagnosis of patients obtained after the review of all clinical folder by two independent observers. Table 2 shows the baseline characteristics of the two subgroups based on the final diagnosis. Patients with aHF had higher BNP and creatinine levels than patients with non-cardiac dyspnea (Tables 2, 3, all *p* < 0.05). Patients with acute HF had more frequent medical history of heart failure and or ischemic heart disease. Table 3 presents mean values of variables considered for diagnostic accuracy in the HF and non-HF populations.

**Table 1:** Final diagnosis by the two independent observers

Final diagnosis	Cases %
Acute heart failure	25 (27)
Pneumonia	13 (14.1)
Acute or exacerbation of COPD/asthma	25 (27.2)
Pleural effusion	9 (9.8)
Pulmonary embolism	1 (1.1)
Primary or secondary lung cancer	12 (13)
Pulmonary fibrosis	2 (2.2)
Anemia	2 (2.2)
Asthma	2 (2.2)
Anxiety	1 (1.1)
Identification of acute heart failure	
Acute heart failure	25 (27)
Non-cardiac dyspnea	67 (73)

**Table 2:** Baseline characteristics and clinical findings detected at the time of patient presentation in the Emergency Department

	Acute heart failure (*n* = 25)	Non-cardiac dyspnea (*n* = 67)	*p*
Age (years)	73.9 ± 13.6	69.35 ± 13.5	0.90
Sex (female)	24%	39%	0.185
Medical history of chronic obstructive pulmonary disease	52%	43.3%	0.455
Medical history of heart failure and/or ischemic heart disease	52%	4.5%	0.0001
Heart rate (bpm)	81.9 ± 22.5	87.9 ± 17.2	0.342
Respiratory rate (breaths/min)	22.7 ± 7	22.5 ± 7	0.921
Systolic BP (mm Hg)	150.6 ± 28	146.1 ± 24.8	0.490
Diastolic BP (mm Hg)	84.1 ± 17.5	82.1 ± 16	0.613
Serum creatinine (mg/dL)	1.46 ± 0.73	0.93 ± 0.41	0.001
White blood cell count (× 10^3^/μL)	9.5 ± 5.45	11.14 ± 4.65	0.19
Oxygen saturation (%)	92.11 ± 6.1	93 ± 6.3	0.791
PaO2/FiO2	291.6 ± 91.26	271 ± 84	0.404
pH	7.43 ± 0.05	7.42 ± 0.06	0.362
Lactate level (mmol/L)	1.65 ± 1.28	1.70 ± 1.09	0.854
C reactive protein	38.74 ± 68	56.9 ± 90	0.312
Procalcitonin	0.45 ± 1.46	2.14 ± 10.7	0.521

**Table 3:** Mean values of variables considered for diagnostic accuracy in the aHF and non-aHF populations

	Acute heart failure (*n* = 25)	Non-cardiac dyspnea (*n* = 67)	*p*
Positive chest X-ray (%)	40	22	0.07
Dilated left atrium (%)	96	33	0.001
Bilateral IS and/or effusion (%)	80	30	0.001
EF ≤ 40 (%)	48	5	0.0001
IVC dilated and not collapsing (%)	65	0	0.0001
BNP	1115 ± 883	169.69 ± 264.4	0.0001
E wave velocity (cm/sec)	99.2	66.9	0.0001
E/A	2.27	0.8	0.0001

Table 4 shows diagnostic performance of all ultrasound parameters, chest X ray and BNP for the diagnosis of acute HF. Chest X-ray and BNP had the lowest accuracy for the identification of acute HF (58.4% and 69%, respectively), significantly lower compare to E/A ratio (both *p* < 0.05). LUS positivity for bilateral IS and/or effusion exhibited high sensitivity (80%), good specificity (70%) and accuracy (76.11%) for the diagnosis of aHF. Dilated left atrium showed the highest sensibility in identifying aHF (96%). In an additional analysis we calculate, through the Yuoden Index, the best cut-off value for E/A ratio to identify acute HF. The Youden Index value for E/A ratio was 0.95. Table 5 shows diagnostic performance of E/A ratio best cut-off values. E/A ratio > 0.95 has higher sensitivity compare to BNP, Chest X-ray and EF and higher specificity compare to dilated left atrium and LUS (Table 5, all *p* < 0.05).

**Table 4:** Sensitivity, specificity, positive and negative predictive value and accuracy of the main diagnostic features

Parameter	Sensitivity (%) (95% CI)	Specificity (%) (95% CI)	PPV (%) (95% CI)	NPV (%) (95% CI)	Accuracy (%)
BNP	88 (68–97)	61 (48–74)	49 (40- 58)	92 (80–97)	69^**†**^
Chest X-ray	40 (22–61)	78 (66–87)	41 (23–63)	77 (65–86)	58.4
Dilated left atrium	96 (78–99)	67 (54–78)	52 (37–67)	98 (87–99)	81.1^*,**†**^
Bilateral IS and/or effusion	80 (57–92)	70 (58–80)	50 (34–66)	90 (78–96)	81.5^*,**†**^
EF ≤ 40	48 (31–65)	95 (82–98)	80 (58–89)	82 (73–84)	71.2^**†**^
IVC dilated and not collapsing	65 (32–69)	98 (91–99)	99 (74–1)	86 (78–95)	83.2^*,**†**^

*p < 0.05 vs NT-proBNP

†*p* < 0.05 vs Chest X-ray

**Table 5:** Sensitivity, specificity, positive and negative predictive value and accuracy of E/A ratio best cut-off values

Parameter	Sensitivity (%) (95% CI)	Specificity (%) (95% CI)	PPV (%) (95% CI)	NPV (%) (95% CI)	Accuracy (%)
E/A > 0.95	88%^*,†,^^−^ (69–97)	85%^*,^^−^^,++,‡^ (82–97)	69% (55.00–80)	95.00% (880–98)	86.87^*,†^

^*^p < 0.05 vs BNP

^†^*p *< 0.05 vs Chest X-ray

-*p* < 0.05 vs EF

+  + *p* < 0.05 vs Dilated left atrium

^‡^*p* < 0.05 vs Bilateral IS and/or effusion

**Fig. 1:**
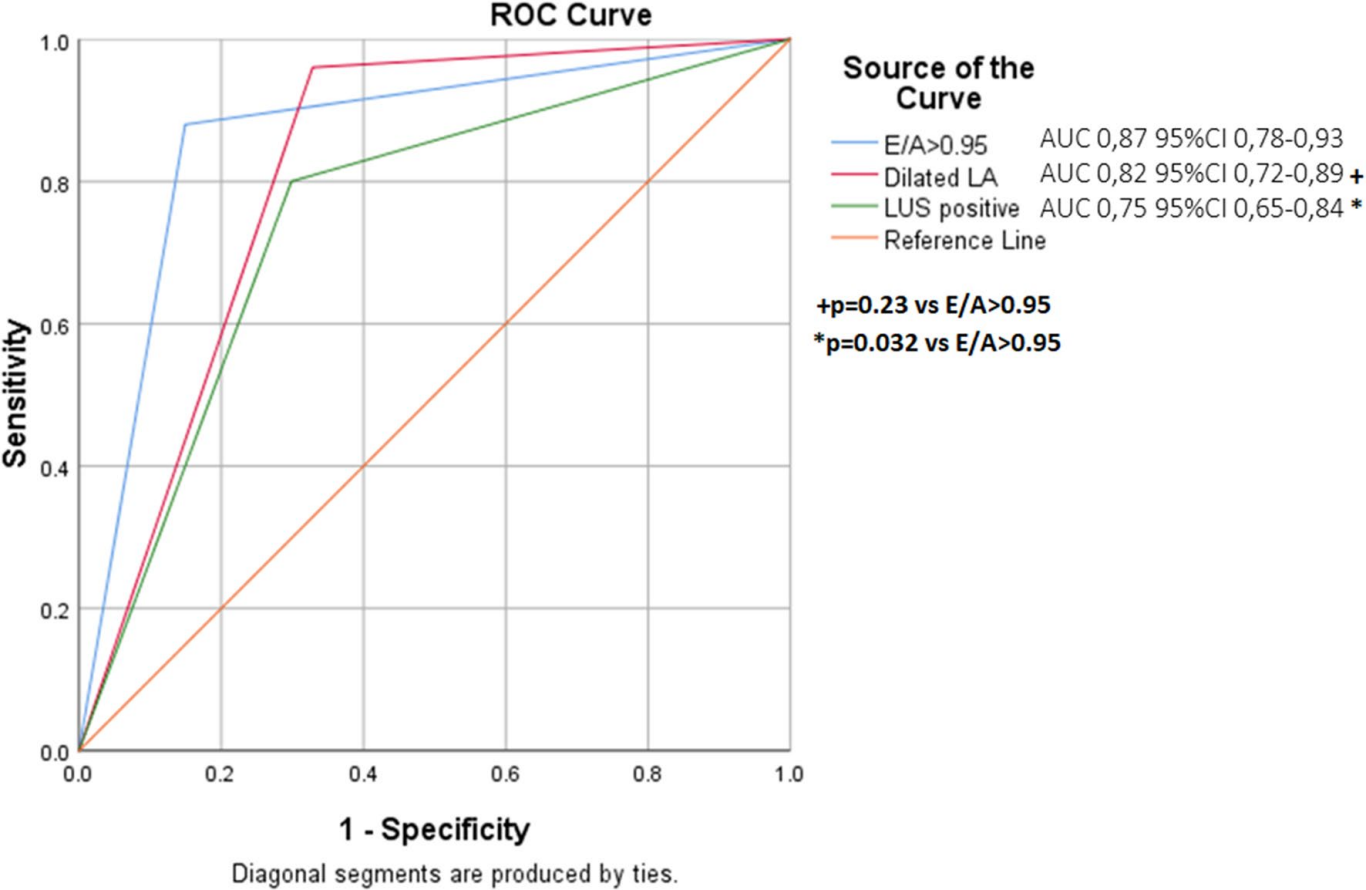
Receiver operating characteristic (ROC) curve comparing accuracy of different ultrasound modalities

**Fig. 2:**
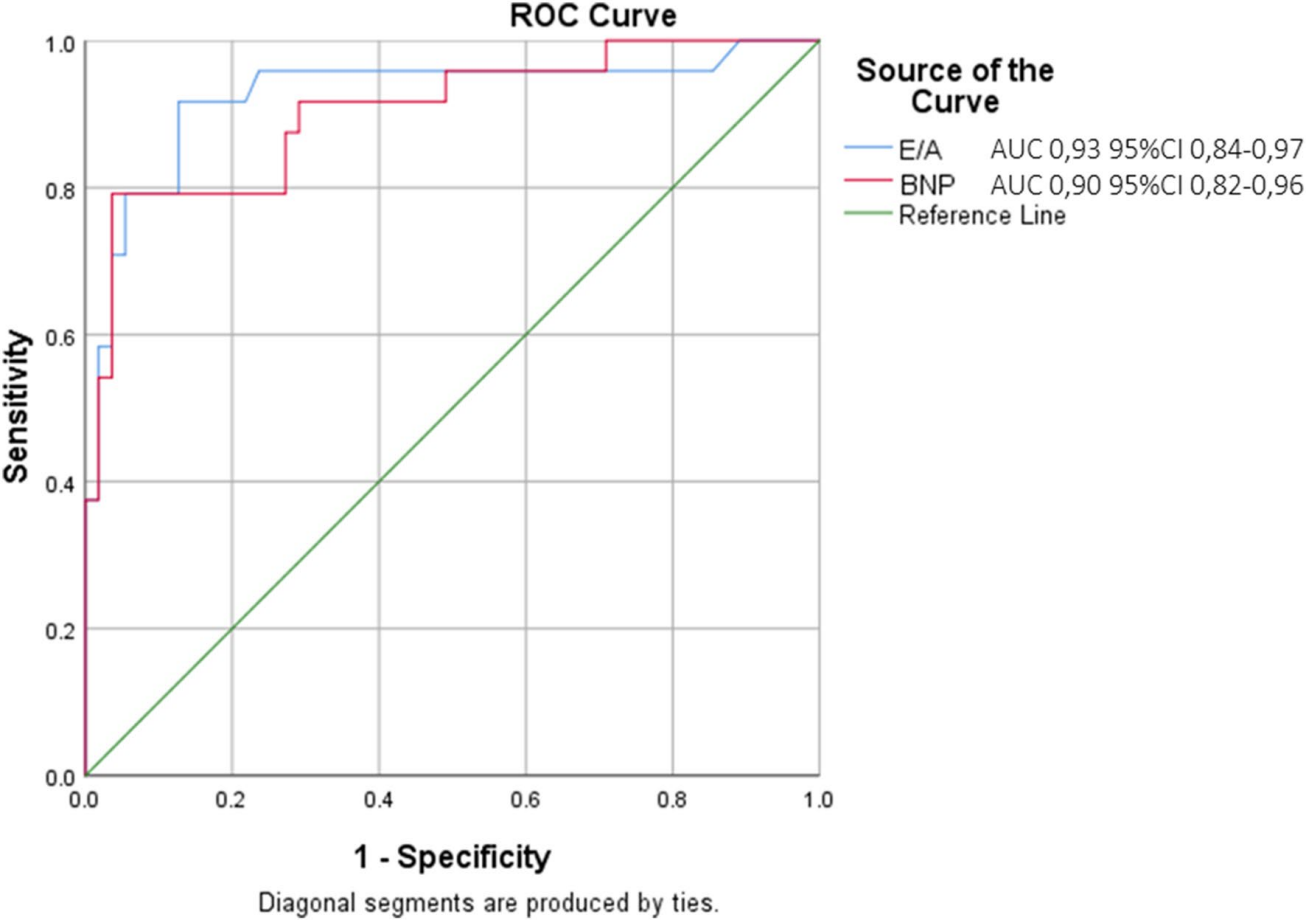
Receiver operating characteristic (ROC) curve comparing accuracy of E/A and BNP level

Roc curve analysis were built to assess diagnostic accuracy of different modalities. Figure 1 shows the area under the ROC curve (AUC) of binary parameters analyzed including best cut-off value of E/A, dilated LA and LUS positivity. E/A > 0.95 showed the highest diagnostic performance with an AUC in identifying aHF of 0.87, significantly higher than LUS (*p* = 0.03). A second ROC curve analysis was built comparing accuracy of E/A ratio with BNP level as continuous variable and is reported in Fig. 2. Compared to NT pro BNP value, E/A ratio showed similar accuracy for the diagnosis of acute HF (0.93 vs 0.90, *p* = 0.69).

**Fig. 3:**
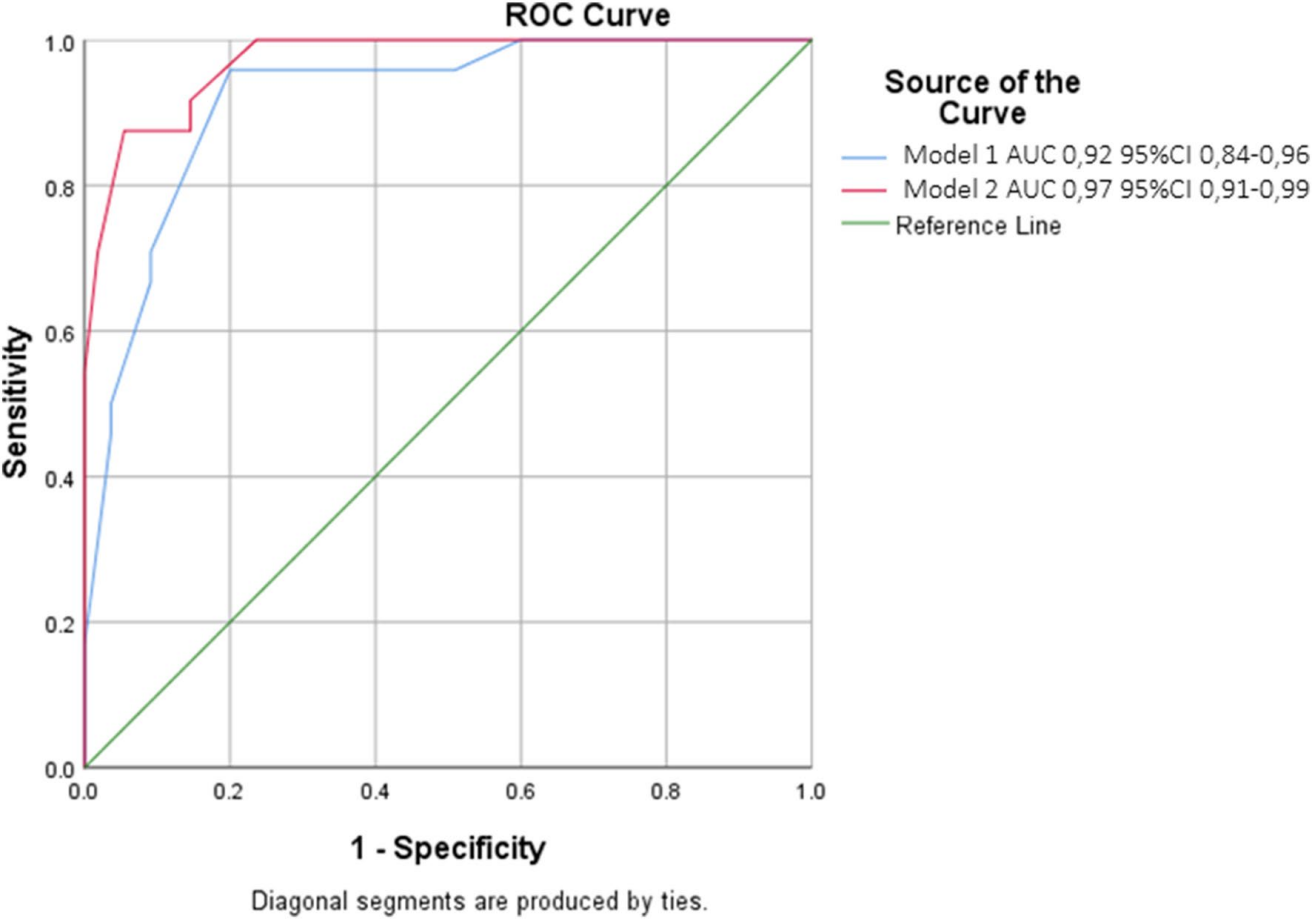
Receiver operating characteristic (ROC) curve comparing accuracy of Clinical score (model 1) and clinical score + E/A ratio (model 2)

Multivariate regression models to predict aHF were built including anamnestic history of heart failure/ ischemic heart disease, chest X-ray, BNP value and dilated LA as model 1. Then, E/A > 0.95 was added in model 2. Figure 3 reports individual hazard functions generated and compared using receiving operating characteristic curves for models 1 and 2. Accuracy of model 2 was significantly higher than model 1 (AUC 0.92 (95%CI 0.84–0.96) vs 0.97(95%CI 0.91–0.99), *p* < 0.02).

## Discussion

The present study demonstrated that the evaluation of E/A ratio in a fast-integrated ultrasound protocol has an excellent accuracy for diagnosis of acute HF in patients presented to the ED with acute dyspnea. E/A ratio is easy to obtain within a 3-min fast ultrasound protocol allowing the identification of patients with acute HF. Our results demonstrated that E/A ratio used on top of clinical examination including anamnestic history, chest X-ray, BNP value and dilated LA significantly improves accuracy in detection of acute HF as a cause of acute dyspnea. Our results strongly encourage the use of E/A ratio as an easy and rapid tool for the diagnosis of HF.

The diagnosis of HF in patients presenting to the ED with acute dyspnea remains challenging. It was demonstrated the utility of integrated heart and lung ultrasound examination for an ultrafast diagnosis of HF in the emergency setting [2, 3]. The main pathophysiological determinant dyspnea in acute HF is increased pulmonary wedge pressure which causes lung interstitial and alveolar edema [27]. LUS is accurate in diagnosing alveolar-interstitial syndrome [28]. The B lines pattern that configures the framework of an alveolar-interstitial syndrome may reflect the presence of either cardiogenic or inflammatory edema, or fibrosis [18]. Pleural effusion may instead be the result of inflammation of the lung parenchyma or heart failure with increased central venous pressure [29, 30]. For this reason, the presence of bilateral IS/effusion alone has high sensitivity as already demonstrated in previous studies but a suboptimal specificity as also observed in the present study [28].

FoCUS identifies cardiac abnormalities and can help to explain the acute onset of HF. Diastolic dysfunction of the left ventricle is presented in all types of HF, regardless of the value of EF. It represents a combination of impaired LV relaxation, restoration forces, myocyte lengthening load, and atrial function, culminating in increased LV filling pressures with congestion or pulmonary edema and left atrial dilation [31]. We have recently demonstrated that left atrial dilatation has an excellent accuracy for the identification of patients with acute HF when combined with LUS exhibiting wet lungs [13]. However, LA dilatation alone, reflecting a chronic diastolic dysfunction, represents a parameters with good sensitivity but suboptimal specificity.

Current Doppler echocardiography guidelines recommend using early to late diastolic transmitral flow velocity (E/A) to assess diastolic function, and E to early diastolic mitral annular tissue velocity (E/e’) to estimate LV filling pressures [23]. Unfortunately, in the Emergency setting, full assessment of diastolic function is not always feasible.

As reported in Table 3, increased E/A ratio is a main characteristic of patient with aHF strongly demonstrating that diastolic dysfunction, even if assessed by simply mitral power Doppler, is a main feature able to identify aHF. Higher E/A ratio is associated with higher LV filling pressure which is the main determinant of development of pulmonary venous congestion [32]. In our study, E/A ratio showed higher specificity then dilated left atrium allowing to extend the spectrum of recognizable acute HF also in patients without dilated left atrium.

In this study, the operator was experienced in both FoCUS and LUS. It was demonstrated that emergency physicians were able to perform and interpret focused echocardiography reliably after a short duration of training [33, 34].

Our study has some limitations. First, the population sample is limited. A larger study population is needed to confirm our results and the tendency of E/A ratio to be statistically more significant than dilated left atrium. It should be underlined that different pathophysiological conditions such as severe mitral annular calcification, severe valvular diseases, stimulated pacemaker rhythm, tachycardia or severe anemia might have impact accuracy of diastolic dysfunction evaluated by E/A ratio. Also, full assessment of diastolic dysfunction is not always feasible and as demonstrated in our study, even simple E/A ratio was not available in about 20% of the study participant [35]. The operators in this study were experienced in both echocardiography and LUS. Nevertheless, echo and LUS can both be learned with a steep learning curve [33]. The use of a simplified protocol for LUS, even if time saving, might have partially influenced our results, in particular reducing sensitivity due to an increase in false negative cases. In this context, E/A ratio appears to be a more diagnostic accurate tool for patients with undifferentiated dyspnea [28]. The clinical score used in the multivariate regression model would have needed a dedicated validation in a successive cohort of patients that we are not able to provide at this stage. However, the main objective of the present study is not to validate a score to diagnose aHF but demonstrated that the use of E/A ratio is helpful and improves diagnostic accuracy of the emergency physician.

## Conclusions

Integrated ultrasound examination is a useful tool for differential diagnosis in patients admitted to the ED with acute dyspnea. In all types of heart failures regardless of ejection fraction, the left ventricle diastolic dysfunction plays an important role in congestion or pulmonary edema and consequent respiratory symptoms. E/A ratio showed an excellent diagnostic accuracy in diagnosing acute HF when assessed in ultrafast ultrasound protocol.

## Data Availability

Details of the study design, statistical analysis plan, and raw data are available on request to the corresponding author.
